# AI-Driven Integrated System for Burn Depth Prediction With Electronic Medical Records: Algorithm Development and Validation

**DOI:** 10.2196/68366

**Published:** 2025-08-15

**Authors:** Md Masudur Rahman, Mohamed El Masry, Surya C Gnyawali, Yexiang Xue, Gayle Gordillo, Juan P Wachs

**Affiliations:** 1 Edwardson School of Industrial Engineering, Purdue University West Lafayette, 315 N Grant Street, West Lafayette, IN, 47907, United States, 1 765 496 7380; 2 McGowan Institute for Regenerative Medicine, University of Pittsburgh, Pittsburgh, PA, United States; 3 Department of Surgery, School of Medicine, University of Pittsburgh, Pittsburgh, PA, United States; 4 Department of Computer Science, Purdue University West Lafayette, West Lafayette, IN, United States; 5 Department of Plastic Surgery, School of Medicine, University of Pittsburgh, Pittsburgh, PA, United States

**Keywords:** burn diagnosis, vision-language model, large language model, ultrasound, electronic medical record, EMR

## Abstract

**Background:**

Burn injuries represent a significant clinical challenge due to the complexity of accurately assessing burn depth, which directly influences the course of treatment and patient outcomes. Traditional diagnostic methods primarily rely on visual inspection by experienced burn surgeons. Studies report diagnostic accuracies of around 76% for experts, dropping to nearly 50% for less experienced clinicians. Such inaccuracies can result in suboptimal clinical decisions—delaying vital surgical interventions in severe cases or initiating unnecessary treatments for superficial burns. This diagnostic variability not only compromises patient care but also strains health care resources and increases the likelihood of adverse outcomes. Hence, a more consistent and precise approach to burn classification is urgently needed.

**Objective:**

The objective is to determine whether a multimodal integrated artificial intelligence (AI) system for accurate classification of burn depth can preserve diagnostic accuracy and provide an important resource when used as part of the electronic medical record (EMR).

**Methods:**

This study used a novel multimodal AI system, integrating digital photographs and ultrasound tissue Doppler imaging (TDI) data to accurately assess burn depth. These imaging modalities were accessed and processed through an EMR system, enabling real-time data retrieval and AI-assisted evaluation. TDI was instrumental in evaluating the biomechanical properties of subcutaneous tissues, using color-coded images to identify burn-induced changes in tissue stiffness and elasticity. The collected imaging data were uploaded to the EMR system (DrChrono), where they were processed by a vision-language model built on GPT-4 architecture. This model received expert-formulated prompts describing how to interpret both digital and TDI images, guiding the AI in making explainable classifications.

**Results:**

This study evaluated whether a multimodal AI classifier, designed to identify first-, second-, and third-degree burns, could be effectively applied to imaging data stored within an EMR system. The classifier achieved an overall accuracy of 84.38%, significantly surpassing human performance benchmarks typically cited in the literature. This highlights the potential of the AI model to serve as a robust clinical decision support tool, especially in settings lacking highly specialized expertise. In addition to accuracy, the classifier demonstrated strong performance across multiple evaluation metrics. The classifier’s ability to distinguish between burn severities was further validated by the area under the receiver operating characteristic: 0.97 for first-degree, 0.96 for second-degree, and a perfect 1.00 for third-degree burns, each with narrow 95% CIs.

**Conclusions:**

The storage of multimodal imaging data within the EMR, along with the ability for post hoc analysis by AI algorithms, offers significant advancements in burn care, enabling real-time burn depth prediction on currently available data. Using digital photos for superficial burns, easily diagnosed through physical examinations, reduces reliance on TDI, while TDI helps distinguish deep second- and third-degree burns, enhancing diagnostic efficiency.

## Introduction

Burn injuries are among the most challenging conditions in medical treatment [1] due to the critical need for accurate depth assessment to determine appropriate therapeutic strategies. Traditional burn depth assessment relies heavily on visual inspection by experienced surgeons, which, while effective in many cases, can lead to inaccuracies, especially when distinguishing between second-degree deep and third-degree burns (76% accuracy for experts, which reduced to 50% for nonexperts) [2-4]. These errors can lead to improper care, postponing critical treatment for severe burns or prompting excessive procedures for minor ones [2,3,5].

**Figure 1.**
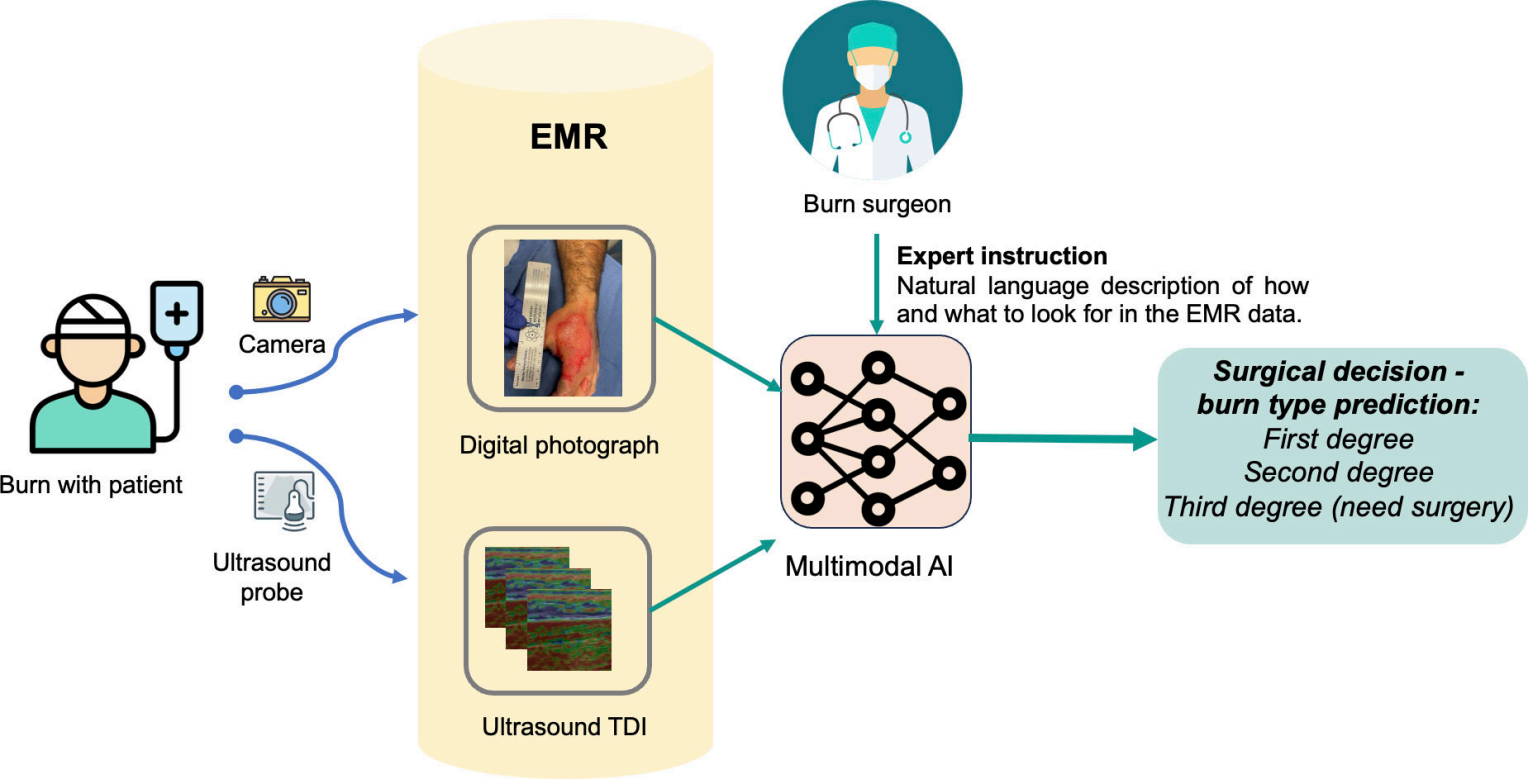
Overview of the proposed multimodal burn classification system, BURN-AID. The framework integrates digital photographs and ultrasound TDI of burn wounds, both stored within an EMR system. A vision-language AI model, guided by expert instructions, analyzes the multimodal data to predict burn severity—classifying injuries as first, second, or third degree. The system supports real-time clinical decision-making by embedding predictions directly into the EMR, enabling streamlined diagnostic workflows, and aiding surgical intervention planning. AI: artificial intelligence; BURN-AID: Burn Diagnosis with Artificial Intelligence; EMR: electronic medical record; TDI: tissue Doppler imaging.

To address these challenges, this paper presents a novel approach, BURN-AID (Burn Diagnosis with Artificial Intelligence), leveraging multimodal imaging integrated with a vision-language model, designed to improve the accuracy of burn depth classification (Figure 1). This system performs a 3-way classification of burn depth into first-, second-, and third-degree burns, offering a more nuanced and precise clinical decision support tool.

The method integrates digital photographs and ultrasound tissue Doppler imaging (TDI) data stored within an electronic medical record (EMR) system. The initial phase of burn assessment uses digital photographs of the burn wound. These images are processed to rapidly classify burns as first degree or second degree [6]. This swift identification is crucial for initiating immediate treatment and reducing the need for further diagnostic procedures for mild burns. However, for cases that potentially classify as third degree, the system relies on TDI data. Ultrasound TDI offers detailed imaging of tissue structures, critical for accurate depth assessment where visual inspection alone may fall short.

The artificial intelligence (AI)–driven approach ensures that ultrasound resources are used judiciously, minimizing unnecessary scans and focusing them on cases with high severity, as indicated by initial digital photograph analysis. This method optimizes the diagnostic workflow within the EMR, allowing for efficient data retrieval and processing, thus enhancing the overall efficiency of the medical staff.

In this study, we present and evaluate BURN-AID, an AI-based method that classifies burn depth using digital photographs and ultrasound-TDI data, measuring its performance across first-, second-, and third-degree categories using expert-annotated clinical datasets.

## Methods

### Recruitment

In total, 30 patients participated in the study, with 1 excluded due to withdrawal. Enrollment was based on a first-come, first-served basis. Patients were screened at the Eskenazi Burn Center, Indianapolis, and informed about the study procedures, time commitment, and risks by the coordinator. Those who qualified signed an institutional review board (IRB)–approved informed consent form.

### Data Exclusion

Patients were excluded if they were unable to provide informed consent, were younger than 18 years of age, had burns covering 75% or more of their body surface, or had burns resulting from chemical, electrical, or radiation sources. Patients with burns that had already undergone surgical debridement were also excluded.

### EMR System

We relied on an EMR system, the DrChrono by EverHealth [7]. We were given access to a training account for medical professionals, allowing us to upload images of burn injuries. We created an app where our AI system can retrieve data from the EMR system and make real-time predictions. Our system was developed in Python (Python Software Foundation) using the Developer API (application programming interface) by DrChrono. Our system is based on a Web API, making it easily extendable to mobile or tablet apps. This integration opens new possibilities for deploying our system in real-time burn diagnosis. Expanding the system to support telemedicine apps could further increase accessibility for patients in remote or underserved areas.

### Algorithm and AI Integration

The method for predicting burn depth integrates multiple data sources and advanced AI algorithms to enhance diagnostic accuracy. The system uses digital photographs and TDI stored within the EMR framework. This multimodal approach leverages the strengths of each data type to provide a comprehensive assessment of burn depth.

### Integration Within EMR Workflows

The AI model is integrated within the EMR system through a custom-built app that uses the Developer API. In practice, the workflow proceeds as follows: when a clinician uploads a new case—including digital photographs and ultrasound images—into the EMR system, our app retrieves this data via the API in real time. The AI model then processes the images, classifies the burn severity using the BURN-AID algorithm (Textbox 1), and returns the prediction directly into the patient’s record within the EMR interface.

**Textbox 1: BURN-AID (Burn Diagnosis with Artificial Intelligence) algorithm.**

1. Start by N observations.
2. Use K for setting parameters. Remains M = N - K.
3. For M, apply the following:
  - Use a classifier Ultrasound-Classifier to determine Degree 3 from non Degree 3.
  - Use a classifier DigitalPhoto-Classifier to determine Degree 1 or Degree 2 or Degree 3.
  - Final Prediction:
    - If Ultrasound-Classifier predicts Degree 3, then it is a Degree 3.
    - If Ultrasound-Classifier predicts non-Degree 3, then take the prediction from the DigitalPhoto-Classifier (highest probability from Degree 1 and 2).
    - If no ultrasound image, then take prediction from DigitalPhoto-Classifier (Degree 1 or Degree 2 or Degree 3).
  - Use ground truth to determine the success rate for every class, and present the results.

This integration ensures minimal disruption to existing clinical workflows. Clinicians continue using the EMR as they normally would, with the added benefit of automated burn severity assessment appearing as part of the patient documentation. The AI-generated predictions are presented in a structured format that can be reviewed, verified, and incorporated into decision-making. Furthermore, since the system is Web API–based, it is platform-agnostic and can be extended to mobile or tablet-based EMR interfaces used at the bedside or in telemedicine contexts. This seamless interaction supports clinicians without requiring workflow changes, enabling real-time, AI-assisted burn diagnosis within the familiar EMR environment.

### Digital Photograph Analysis

We designed a prompt with expert instructions to be given to the vision-language model to generate burn depth predictions. The detailed process is described as follows:

Several color images were taken for each burn area of the patient, with each image (dimension 4032×3024). Per patient, the number of images was a median of 4 (IQR 0), an average of 4 (SD 0.96), a minimum of 2, and a maximum of 7. For each image of the same burn, our AI system first provides a prediction along with an explanation. Based on the majority of the vote, the model then provides an overall classification. The predictions are based on the type of damage to the skin layers as follows [8,9]:

- Epidermis layer: The thin, outermost layer of the skin containing melanocytes, which produce melanin (skin pigment).
- Dermis layer: The middle layer containing blood vessels, lymph vessels, hair follicles, sweat glands, collagen bundles, fibroblasts, and nerves. It is held together by collagen produced by fibroblasts and contains nerve endings that convey pain and touch signals.
- Hypodermis (subcutaneous) layer: The deepest layer consisting of a network of collagen and fat cells that conserve body heat and protect it from injury.

Definitions based on burn classification given to the AI model are as follows: [10]

- First degree: Redness of the skin without blisters.
- Second degree superficial: Presence of blisters, dermis intact.
- Second degree deep: Dermis is burnt; the exact depth is unknown.
- Third degree: Damage through the dermis with eschar (white, yellow, or black), involving part or all of the hypodermis (subcutaneous layer).

The final classifier consists of a multimodal setting, where ultrasound TDI data are used along with digital photographs. Thus, if TDI data are available, the BURN-AID model first uses the TDI analysis module to generate a binary label: third degree or nonthird degree. This prediction is given to our AI system to make an overall prediction. In scenarios where TDI data predict third-degree burns, the overall system predicts third-degree burns. When TDI indicates non-third-degree burns, the digital photographs are used for further fine-grained classification, identifying first- and second-degree burns. This system design ensures that less severe burns (first degree) can be classified without needing ultrasound TDI scanning. However, it incorporates TDI data when available. Additionally, our system design integrates EMRs in real time as soon as the data are available in the EMR database for a patient. We further discuss the classification with ultrasound TDI data only.

### TDI Analysis

TDI is an effective technique for evaluating the mechanical response of tissues to externally applied forces by assessing tissue stiffness using a strain gauge incorporated into the ultrasound probe [11-13]. Images are only collected when the application pressure on the probe is within an acceptable range, as detected by the strain gauge. Burn injuries cause the denaturation of proteins, which alters the elastic properties of subcutaneous tissue. TDI is instrumental in detecting these alterations, helping to determine the depth of burn penetration beyond the superficial skin layers into the subcutaneous tissue [11-13]. This is critical for identifying injuries that may require surgical intervention, such as excision and grafting.

TDI produces color-coded images where different hues (blue for hard tissue and red for soft tissue) indicate the mechanical state of the tissue. Changes in these color patterns from healthy skin, due to burn-induced modifications, are crucial for the system’s enhanced diagnostic accuracy. The interpretation of TDI images involves analyzing the distribution of red, green, and blue colors to predict burn depth. Continuous blue lines in the TDI images suggest third-degree burns, as this indicates the tissue has hardened due to the burn.

Burn surgeons formulated a natural language interpretation of TDI images of burn injuries, which were fed into the AI system, as follows: TDI assesses tissue displacement in response to an applied mechanical force, evaluating tissue stiffness. The image can be interpreted vertically from top to bottom, corresponding to the skin’s layers: the epidermis at the top, followed by the dermis, and the hypodermis (subcutaneous layer) at the bottom. Third-degree burns are identified by a predominant, continuous blue pattern in the hypodermis, indicating severe burns that extend through the dermis to the deepest skin layer. Non-third-degree burns lack this dominant blue pattern in the hypodermis, indicating lesser severity.

### BURN-AID Framework Overview

Figure 1 illustrates the workflow of the proposed multimodal burn classification system, BURN-AID. The process begins with the patient with burn, whose injury is documented using both a digital camera and an ultrasound probe. The digital photograph captures surface-level visual information, while the ultrasound probe collects TDI data to assess underlying tissue characteristics.

Both types of data are stored within the patient’s EMR. The AI system retrieves this multimodal data from the EMR and uses it as input for classification. In addition to the image data, the AI model incorporates expert surgeon instructions, formulated as natural language descriptions. These instructions guide the model on what to look for within the EMR to support clinical decision-making.

The AI system, trained to recognize patterns associated with varying burn depths, then outputs a prediction. The final classification determines whether the burn is first degree, second degree, or third degree. Importantly, third-degree burns—those that typically require surgical intervention—are flagged accordingly. This setup enables accurate, real-time diagnostic support directly within the clinical workflow.

### AI Model Implementation

Leveraging a vision-language model, specifically the GPT-4o [14], enables the exploration of patterns described in the expert instruction within images. The AI model uses expert instructions (system prompt), digital photographs, and TDI ultrasound images (when available) to generate explanations in natural language. The output format for assessing burn depth using TDI is structured into 2 components: burn depth, which specifies the assessed depth of the burn, providing direct insight into injury severity; and explanation, which provides a detailed rationale for the prediction, elaborating on how observed color patterns in TDI images correlate with the predefined hypothesis regarding burn depth and severity. When analyzing multiple images, the system provides predictions and explanations for each image first, followed by an overall assessment based on the majority of images.

For our experiments, we implemented the algorithm using Python to encode the natural language expert instructions into the system message prompt. For example, expert instructions are: for a digital photograph, “consider 1st degree burn if there is redness of the skin without blisters,” and for TDI, “consider 3rd degree burn if there is a continuous blue pattern in the hypodermis (lower layer/bottom part of the image).” The OpenAI GPT-4 Vision API [14] was used to submit the expert instruction and request burn depth predictions with the prompt “What is the depth of the burn indicated by this image?” The system provided predictions for a 3-way classification of burn depth: first-, second-, and third-degree burns. The experimental procedure focused on generating predictions and explanations at the per-patient level. By focusing on per-patient evaluation, the system ensured accurate burn depth classification and appropriate surgical decision-making, enhancing the overall efficiency and effectiveness of burn injury diagnosis and treatment.

### Burn Severity Classification With BURN-AID

To classify the severity of burns, we propose a hybrid method called BURN-AID, detailed in Textbox 1. This method integrates 2 classifiers based on different imaging modalities: ultrasound and digital photographs. The algorithm begins with a dataset of N observations. A subset of K observations is used for parameter tuning, leaving M=N−K observations for the main classification process.

For each of the M observations, 2 classifiers are applied. The first is an ultrasound-based classifier that performs a binary classification to distinguish between Degree 3 burns and non-Degree 3 burns. The second is a digital photo–based classifier that performs multiclass classification to differentiate among Degree 1, Degree 2, and Degree 3 burns.

The final prediction for each observation follows a decision hierarchy. If the ultrasound classifier predicts the burn as Degree 3, this prediction is used as the final classification. If the ultrasound classifier predicts a non-Degree 3 burn, the algorithm then relies on the digital photo classifier, selecting the class with the highest probability between Degree 1 and Degree 2. In cases where an ultrasound image is not available, the prediction is made solely based on the output of the digital photo classifier among all 3 classes. After completing the classification for all M observations, the predicted burn degrees are compared with the ground truth labels.

### Dataset Description for Experiments

A major challenge is finding publicly available datasets to conduct experiments in our setting. While some datasets can be found through internet sources, such as Google Image search, these images often lack appropriate annotations and are frequently copyrighted. Additionally, there is no available ultrasound TDI data for second- and third-degree burns. As a result, we rely on data collected in hospital settings.

The dataset used for this study consists of a total of 41 patients, encompassing various degrees of burn severity: 15 patients with first-degree burns, 16 patients with second-degree burns, and 10 patients with third-degree burns. Table 1 shows details of the data statistics.

For fine-tuning the prompt and optimizing the parameters of the proposed classifier, we used data from a subset of 9 patients: 3 with first-degree burns, 3 with second-degree burns, and 3 with third-degree burns. The majority of the data (n=29), which we collected, was gathered in a hospital setting, the Richard M. Fairbanks Burn Center at Eskenazi Hospital, Indianapolis, IN, United States.

**Table 1.** Distribution of patients by burn severity used for evaluating the BURN-AID (Burn Diagnosis with Artificial Intelligence) system (N=41).

Item	Values, n (%)
First-degree burn	15 (37)
Second-degree burn	16 (39)
Third-degree burn (need surgery)	10 (24)

### Validation

The digital photograph was captured first, immediately followed by TDI scanning to ensure that both modalities refer to the same stage of the burn. Two individuals were involved in the data collection process to ensure consistency and efficiency.

However, for a balanced representation, we included data from 12 patients with first-degree burns obtained from a previously published burn dataset [15], as first-degree burn cases typically heal at home and thus are underrepresented in hospital.

To establish the ground truth, 2 burn specialists (burn surgeons) independently and blindly assessed each image, reviewing multiple angles to inform their decisions. On average, each expert took approximately 2 minutes per case. When initial assessments differed, they collaborated to resolve discrepancies through a consensus process, which took up to 3 minutes per case. This final consensus served as the reference standard, against which we compared the validation results to evaluate the accuracy and reliability of our method.

To ensure a comprehensive evaluation, the classifier was assessed using a held-out set of 32 patients, comprising 12 with first-degree burns, 13 with second-degree burns, and 7 with third-degree burns. This diverse dataset allows for a robust analysis of the classifier’s performance across different burn severity levels.

### Statistical Analysis

Accuracy measures the proportion of correctly classified instances out of the total instances. The *F*_1_-score is particularly useful in situations where the class distribution is imbalanced, as it is the harmonic mean of precision and recall, offering a single metric that balances both concerns. Specificity was used to measure the classifier’s ability to correctly identify negative cases (ie, not classifying an instance as a burn type when it is not). The area under the receiver operating characteristic (AUROC) was used to demonstrate the classifier’s ability to discriminate between different classes. AUROC values close to 1 indicate excellent discrimination ability, suggesting that the classifier is very effective at distinguishing between different classes. The confusion matrix provides a detailed breakdown of the classifier’s performance by showing the number of true positive, true negative, false positive, and false negative predictions. To assess the statistical significance of the observed accuracy, a permutation test with 10,000 permutations was conducted, where a low *P* value indicates a low probability that the observed accuracy could have been achieved by random chance.

### Ethical Considerations

The study protocol and experimental design were reviewed and approved by the IRB (IRB# 12689) at Indiana University. All patients provided informed consent to participate and were compensated (up to US $300 per patient: US $100 for the first session and US $50 for each subsequent session) in accordance with the IRB protocol and the ethical principles outlined in the Declaration of Helsinki. To protect the privacy and confidentiality of research subjects, all data were deidentified (anonymized) prior to analysis.

## Results

This study evaluated the performance of a multimodal AI system designed to identify first-, second-, and third-degree burns from images stored in an EMR. The classifier’s performance was assessed using several metrics to ensure both effectiveness and reliability. Below, we present the performance metrics.

### Evaluation Outcomes

#### Accuracy

The classifier achieved an accuracy of 84.38%. Accuracy is the proportion of correctly classified (aligned with the decision made by clinicians) instances out of the total instances. The software was least accurate with first-degree burns, for example, sunburns, likely because the thermal injury does not induce any changes below the epidermis, so TDI images will show little change, as this is a small contributor to skin elasticity relative to the dermis. Fortunately, this is also the easiest burn depth to diagnose clinically, and digital photos are usually sufficient.

#### *F*_1_-Score

**Figure 2.**
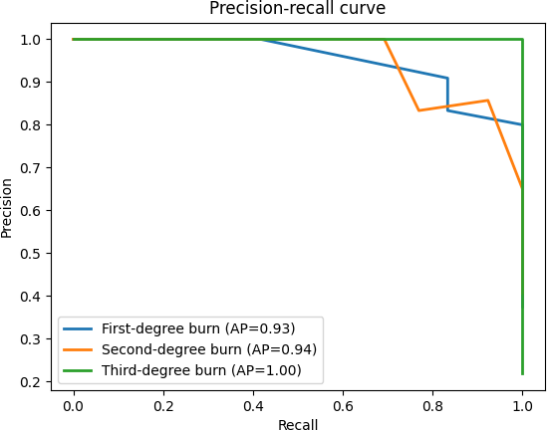
Precision-recall curves for each burn severity class—first, second, and third degree—demonstrating the performance of the BURN-AID classifier in distinguishing between burn types. The curves highlight the trade-off between precision and recall, with high area under the curve values indicating strong predictive accuracy, particularly for third-degree burns. These results support the model’s robustness in identifying clinically significant burn injuries with high confidence. AP: average precision; BURN-AID: Burn Diagnosis with Artificial Intelligence.

The *F*_1_-score, which provides a balance between precision and recall, was 0.856. The precision-recall curve is given in Figure 2, showing the trade-off.

#### Specificity

The specificity, representing the proportion of true negative (nonsurgery case classified as nonsurgery) results among all negative cases, was 83.33%.

#### Area Under the Receiver Operating Characteristic Curve

**Figure 3.**
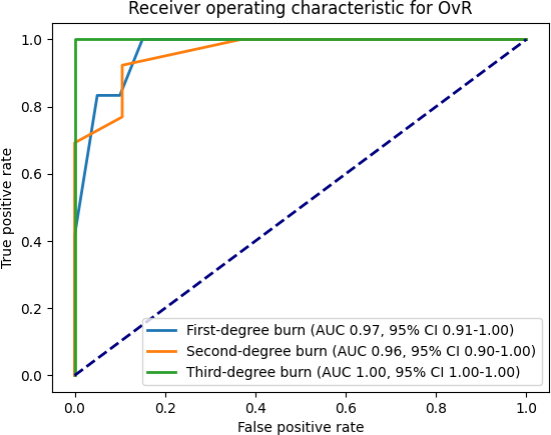
AUROC for OvR classification of first-, second-, and third-degree burns using the BURN-AID system. The AUC scores indicate high discrimination ability for each class, with near-perfect performance for third-degree burns (AUC 1.00). The narrow CIs reflect the model’s consistency and reliability in differentiating burn severities, supporting its utility in clinical decision-making. AUC: area under the curve; AUROC: area under the receiver operating characteristic; BURN-AID: Burn Diagnosis with Artificial Intelligence; OvR: one-vs-rest.

The plot is given in Figure 3, which illustrates the trade-off between the true positive rate and the false positive rate for each class. The AUROC curve for the one-vs-rest approach was 0.97 (95% CI 0.91‐1.00) for first-degree burns, 0.96 (95% CI 0.90‐1.00) for second-degree burns, and 1.00 (95% CI 1.00‐1.00) for third-degree burns, indicating excellent discrimination ability with high AUROC values (Figure 3). The 95% CI range for each class reflects the precision of the AUROC estimate, with narrower ranges indicating more reliable estimates. For instance, the third-degree burns have the narrowest CI (1.00‐1.00), indicating a highly reliable AUROC estimate, whereas the first- and second-degree burns have wider CIs, suggesting a bit more variability in the estimate. These high AUROC values and their respective CIs suggest that our method is effective at distinguishing between the different classes. This demonstrates that the proposed classifier performs well across all classes, with the high AUROC and narrow CI range highlighting its robustness in a multiclass setting.

#### Confusion Matrix

**Figure 4.**
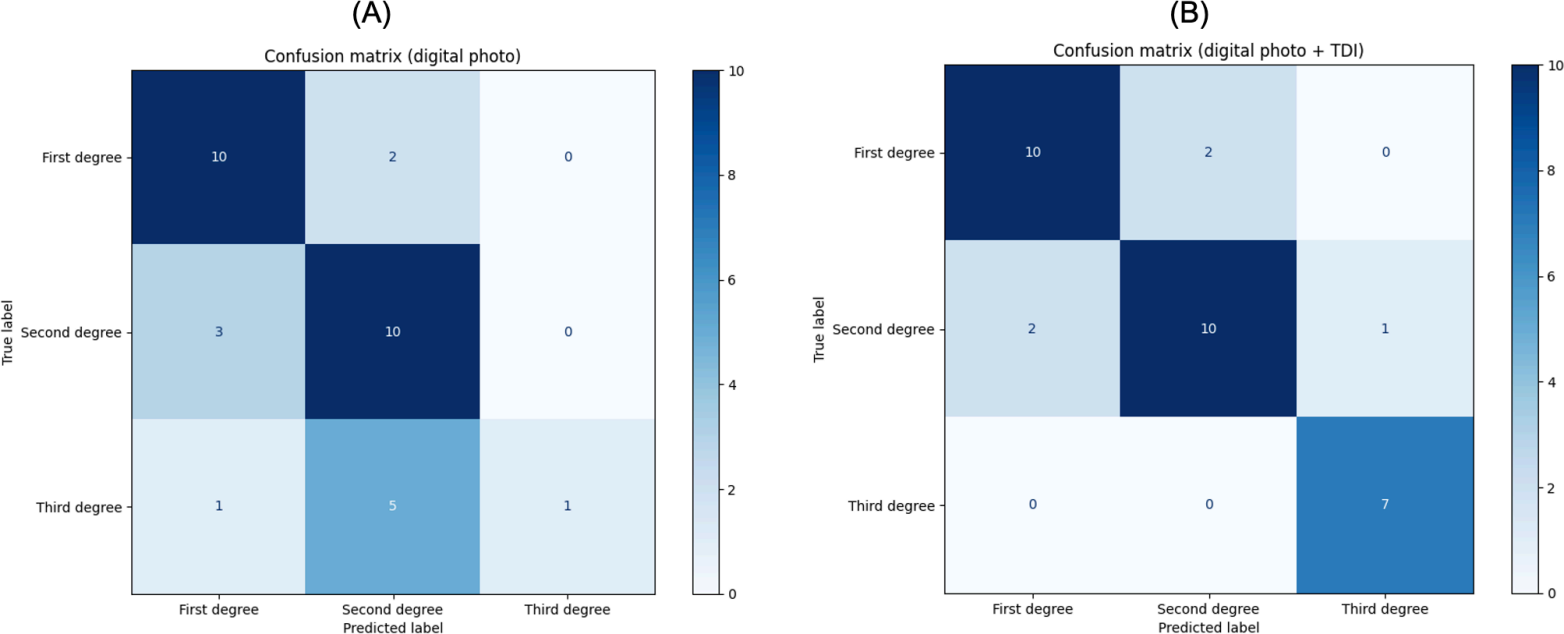
Confusion matrix visualizations showing the performance of the BURN-AID classifier across 3 burn severity levels. (A) Using digital photographs only, the model correctly classified most cases of less severe burns (between first and second degree) but frequently misclassified second-degree burns as third degree, potentially overestimating severity. (B) With both digital photographs and TDI, the classifier achieved its highest accuracy for third-degree burns and showed improved overall performance. These results demonstrate the advantage of multimodal imaging in improving diagnostic accuracy, particularly for identifying severe burns that require surgical intervention. BURN-AID: Burn Diagnosis with Artificial Intelligence; TDI: tissue Doppler imaging.

The confusion matrix (Figure 4A and B) provides a detailed breakdown of the classifier’s performance by showing the number of true positive, true negative, false positive, and false negative predictions. For first-degree burns, the classifier correctly identified 10 cases, with 2 misclassified as second-degree burns; for second-degree burns, 10 cases were correctly identified, with 2 misclassified as first-degree burns and 1 as a third-degree burn; and for third-degree burns, all 7cases were correctly identified with no misclassifications. The confusion matrix visualization provides a detailed view of the classifier’s performance across the different burn degrees.

### Significance of the Performance

A permutation test with 10,000 permutations was conducted to assess the statistical significance of the observed accuracy of the classifier. The *P* value was found to be .0001, indicating a very low probability that the observed accuracy could have been achieved by random chance. This reinforces the conclusion that the classifier is performing significantly better than a random classifier.

## Discussion

The goal of this research is to create an integrated multimodal AI system within an EMR framework that enhances burn depth classification accuracy while optimizing diagnostic precision and resource use.

### Principal Findings

Our method achieves an accuracy of 84% that correlates with the decision of expert burn surgeons regarding burn depth prediction. The AI system we developed for burn diagnosis provides natural language explanations that have the potential to assist clinicians in making informed decisions. Unlike other deep learning–based methods that use ultrasounds and digital photographs and often offer explanations in the form of saliency maps (eg, LIME [16]), which require further processing to be useful, our system delivers direct natural language text. This approach drastically reduces the gap in explanations for AI systems, making them more accessible to general clinicians. Additionally, in cases of misprediction, these explanations aid in making final decisions, thereby mitigating the risk of incorrect treatment decisions.

### Comparison With Prior Work

Our system’s effectiveness is built on several mechanisms for burn depth diagnosis. Traditional machine learning and deep learning models [17,18] typically require extensive training data to make accurate predictions, often needing millions of samples to learn useful features from images [19-23]. However, this requirement limits their application in clinical settings, especially in health care areas like burn diagnosis, where data are limited, difficult to obtain, and ultrasound data are even more challenging to acquire [6,24-27]. In our case, we used a small number of patient data to fine-tune the system for burn diagnosis, achieving reasonable accuracy.

We leverage large-scale pretraining, which does not require burn data. Instead, the system is pretrained to understand general knowledge, such as identifying patterns in images. We also incorporate burn surgeons’ instructions directly into the system as prompts for making predictions. These instructions simplify the decision-making process because the AI system only needs to identify patterns in the ultrasound images as instructed by the ultrasound and surgeon experts. This expert knowledge can be prerecorded, eliminating the need for the expert presence during real-time decision-making. The expert knowledge is embedded in the system training, making it unnecessary during inference or real-time predictions.

The scarcity of publicly available clinical burn images poses a significant challenge for the research community in advancing this field [28]. Several prior works have resorted to using internet-sourced burn images to develop machine learning systems for depth prediction [28]. Digital photos, with careful preprocessing, such as isolating the burn area and resizing images to 224×224 pixels from a single burn wound, are often used [29,30]. In contrast, we consider using a digital photograph of the burn wound with patients’ body parts. This method does not require burn segmentation and is thus more practical in clinical settings. Additionally, our method provides a mechanism to combine digital photography with ultrasound imaging to address errors associated with digital photographs alone, particularly regarding burn depth, which is not visible in standard digital photographs.

Digital photograph–based burn classification has been explored in deciding surgical decision-making for patients with burn [18]. It has been observed that the accuracy in surgical decision-making from digital photographs is 64.7% [18]. This might be due to the fact that the digital photo contains limited information about the deeper skin layers, which are required to identify third-degree burns or surgical cases. In contrast, we leverage TDI data, which improves the accuracy to 84.38% in our AI system. In the literature, various ultrasound methods have been used to diagnose diseases, including burns. Some studies include pig data, use laboratory settings, and achieve reasonable performance [31]. The B-mode ultrasound technique uses a contact probe operating at high frequencies to measure and assess characteristics of skin tissue. In contrast, we build and evaluate our AI system in a multimodal setting, leveraging TDI ultrasound data on human participants. Integration with EMR systems allows our multimodal system to be used in clinical settings where EMR data, such as digital photographs and ultrasound images, can be combined to make comprehensive decisions. This integration reduces costs since some scenarios, like predicting a first-degree burn, might only require digital photographs, eliminating the need for ultrasound. This flexibility helps reduce diagnosis costs and enables faster real-time predictions, which can be updated with new data from the EMR system.

Our system has the potential to be applied to the extensive patient data available in EMR systems across hospitals, requiring minimal involvement from burn experts. Adopting our method to new EMR systems requires generating input data of the burn wound, specifically digital photographs and ultrasound imaging. The AI system then processes this input and provides burn depth information as output. This capability could assist expert surgeons in making more informed decisions and potentially highlight areas where decisions may require further review. The portable nature of imaging systems such as cameras and ultrasound probes allows our system to be used outside hospital settings (eg, accidents, fire incidents, and remote areas). Developing this system for mobile settings, such as tablet and mobile apps, is our next step. Developing such a user-friendly app will be of ultimate importance in far areas where there is no tertiary care or burn centers available.

### Limitations

While the proposed method demonstrates promising results in predicting burn depth using multimodal data, several limitations must be considered. First, the use of digital photographs of burn wounds introduces the potential for bias related to variations in skin color. Since skin tone can affect color perception and image-based classification, this could influence model performance. To mitigate this, we cropped the images to focus exclusively on the wound area, excluding surrounding skin and other body parts, thereby minimizing skin tone as a confounding factor. However, in real-world clinical settings, such precise cropping may not always be feasible. Future work should explore model robustness to a wider range of skin tones and develop techniques that are less sensitive to background features. Second, in the case of TDI, we selectively cropped the images to include only the upper portion where the skin layers were clearly distinguishable. This step improved consistency and clarity in the analysis. Nevertheless, this approach assumes ideal image quality and positioning, which may not hold true in routine clinical environments. As a result, the algorithm’s generalizability to full, uncropped TDI images remains to be fully validated. Future developments could incorporate automated region-of-interest detection or full-image interpretation to better simulate real-world use. Third, the validation of our AI model relied on expert annotation. We implemented a systematic approach involving blind evaluations and consensus discussions between burn surgeons, who also had access to B-model and TDI ultrasound data during annotation. These decisions were informed by actual clinical treatments, providing a strong reference for classification. However, the current clinical gold standard for burn depth assessment remains histological examination via biopsy. In our study, a subset of patients (5/29) had corresponding histology data, which served as definitive ground truth. Although our expert annotations were consistent with these gold-standard cases, the limited histological data constrain the broader validation of our model’s accuracy. Future studies should aim to include more histopathological data to strengthen model evaluation and reduce reliance on subjective expert consensus.

### Conclusions

This study presents BURN-AID, an AI-based system for classifying burn depth using multimodal imaging, digital photographs and ultrasound TDI, integrated within an EMR framework. We evaluated its performance on expert-annotated datasets, achieving an overall classification accuracy of 84% across first-, second-, and third-degree burns. These results demonstrate the potential of multimodal AI to support clinical decision-making in burn diagnosis.

## Abbreviations

AI: artificial intelligence
API: application programming interface
AUROC: area under the receiver operating characteristic
BURN-AID: Burn Diagnosis with Artificial Intelligence
EMR: electronic medical record
IRB: institutional review board
TDI: tissue Doppler imaging
